# Radiative Peristaltic Flow of Jeffrey Nanofluid with Slip Conditions and Joule Heating

**DOI:** 10.1371/journal.pone.0148002

**Published:** 2016-02-17

**Authors:** Tasawar Hayat, Maryam Shafique, Anum Tanveer, Ahmed Alsaedi

**Affiliations:** 1 Department of Mathematics, Quaid-I-Azam University 45320, Islamabad 44000, Pakistan; 2 NAAM Research Group, Department of Mathematics, Faculty of Science, King Abdulaziz University, Jeddah 21589, Saudi Arabia; Tsinghua University, CHINA

## Abstract

Mixed convection peristaltic flow of Jeffrey nanofluid in a channel with compliant walls is addressed here. The present investigation includes the viscous dissipation, thermal radiation and Joule heating. Whole analysis is performed for velocity, thermal and concentration slip conditions. Related problems through long wavelength and low Reynolds number are examined for stream function, temperature and concentration. Impacts of thermal radiation, Hartman number, Brownian motion parameter, thermophoresis, Joule heating and slip parameters are explored in detail. Clearly temperature is a decreasing function of Hartman number and radiation parameter.

## 1 Introduction

Peristaltic activity has great value in many physiological processes and industries. Peristalsis can occur due to contraction and expansion of flexible boundaries. In other words this activity includes passing down, mixing and transporting materials through contraction or expansion of the waves propagating along the channel walls. It has wide applications in medical industry and chemical processes. Typical examples in this direction include in distillation towers and fixed-bed reactors, urine transport from kidney to bladder through the ureter, transport of lymph in the lymphatic vessels, swallowing food through the esophagus, the movement of chyme in the gastrointestinal tract, ovum movement in the fallopian tube, transport of corrosive fluids, sanitary fluid transport and blood pumps in heart lung machine etc. The worms utilize peristalsis for locomotion. Latham [1] and Shapiro et al. [2] initiated works on peristalsis of viscous fluids via theoretical and experimental approaches. Later on many researchers put forward their research on this topic by considering different kinds of fluid models, no-slip/ partial slip condition and one or more assumptions of long wavelength, low Reynolds number, small amplitude ratio, small wave number etc. Especially the magnetohydrodynamics (MHD) peristaltic transport of fluid in a channel are quite important with reference to conductive physiological materials for example the blood, blood pump machines and with the need of both experimental and theoretical research for operation of peristaltic MHD compressor. Concept of magnetohydrodynamics is useful in Magnetic Resonance Imaging (MRI) when a patient undergoes in a height static magnetic field. On the other hand the heat transfer in peristalsis is useful in the oxygenation processes. Such concept in further important in the industrial applications like sanitary fluid transport and transport of corrosive materials where the fluid contact with the machinery parts is prohibited. Note that the heat transfer on skin surface occurs by any of the four processes namely evaporation, convection, conduction and radiation. Having all such aspects in mind many authors in past analyzed the peristaltic flows in detail (see [3–16]). Nadeem and Akbar [17] studied influence of radially varying MHD on the peristaltic flow in an annulus. Ellahi and Hussain [18] analyzed effects of MHD and partial slip on peristaltic flow of Jeffrey fluid in a rectangular duct. Ali et al. [19] analyzed numerical simulation of peristaltic flow of a biorheological fluid with shear dependent viscosity in a curved channel.

Mixed convection occurs in vertical channels for improvement of cooling systems in engineering. Analysis of heat transfer with MHD and mixed convection in vertical channels has great applications in solar energy collection, chemical reactions and cooling systems. Sheikholeslami et al. [20] analyzed simulation of MHD CuO–water nanofluid flow and convective heat transfer using Lorentz forces. Abbasi et al. [21] discussed effects of inclined magnetic field and Joule heating in mixed convective flows of non-Newtonian fluids. Mustafa et al. [22] analyzed Soret and Dufour effects in the mixed convective peristaltic flow of fourth grade fluid. Soret and Dufour effects in mixed convective peristalsis of viscous nanofluids are examined by Hayat et al. [23]. Srinivas and Muthuraj [24] addressed mixed convective peristalsis in presence of chemical reaction. Heat and mass transfer analysis in mixed convective peristaltic transport of viscous fluid in an asymmetric channel is studied by Srinivas et al. [25]. It has been noticed that not much information is available for mixed convective peristalsis of non-Newtonian fluid in a channel especially with compliant walls and nanoparticles. The purpose here is to examine the mixed convective peristaltic flow of Jeffrey nanofluid in a compliant walls channel. In addition thermal radiation effect is considered. The channel walls exhibit velocity, thermal and concentration slip conditions. Joule heating is taken into account. Results for velocity, temperature and concentration are obtained.

Peristaltic transport has not been conducted well in connection with elastic behavior of the walls. Wall properties such as elastic tension and damping are of immense importance in practical circumstances. Graphical results are plotted to analyze the behavior of sundry parameters on temperature, velocity, nanoparticle concentration and heat transfer coefficient.

## 2 Modeling

**Fig 1:**
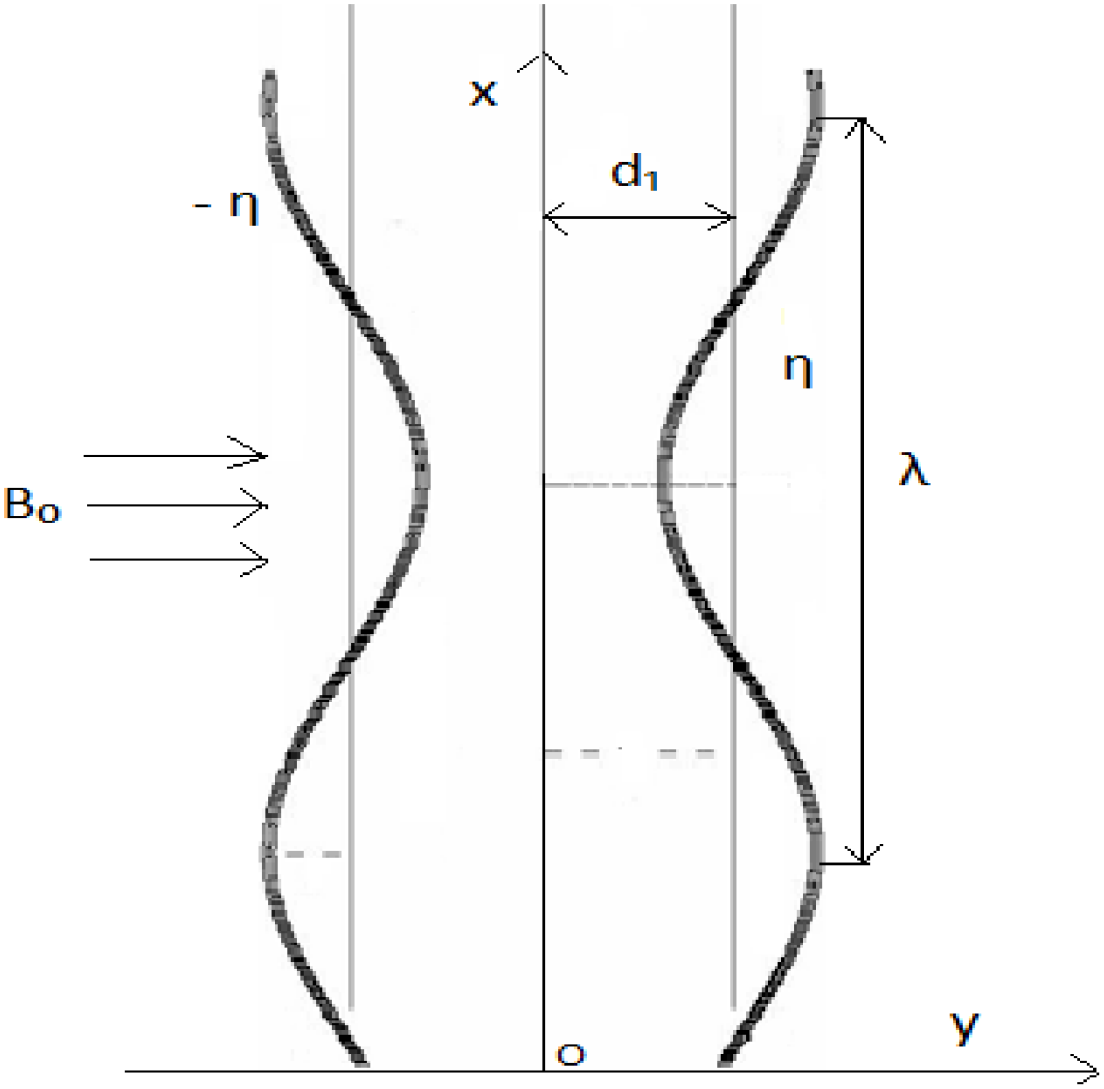
Geometry of the problem.

We consider two-dimensional flow of an incompressible Jeffrey nanofluid in a symmetric channel of uniform thickness 2*d*_1_. The sinusoidal wave is propagating along the walls of the channel with wavelength *λ* and constant speed *c*. The axial and transverse directions are indicated by *x* and *y* respectively. Let *y* = −*η* and *y* = +*η* shows the left and right positions of the channel boundaries (see Fig 1). A magnetic field of strength *B*_0_ is applied. The effects of induced magnetic field is negligible for small magnetic Reynolds number. The slip conditions for velocity, temperature and concentration are considered. The electric field is considered absent. The walls of channel are taken flexible. The wall geometry can be described by the expression

$$y=\pm \eta \left(x,t\right)=\pm \left[d_{1}+a\sin\frac{2\pi }{\lambda }\left(x-ct\right)\right],$$ (1)

where *c* is the wave speed, *a* the wave amplitude, *λ* the wavelength, 2*d*_1_ the width of channel and *t* the time. Expression of Cauchy stress tensor (*τ*) for Jeffrey material is

τ=-pI+S,

$$S=\frac{\mu }{1+\lambda_{1}}\left(\dot{\gamma }+\lambda_{2}\frac{d\dot{\gamma }}{dt}\right).$$

Here **S** represent extra stress tensor, *λ*_1_ shows the ratio of relaxation to retardation times, *λ*_2_ the retardation time, *p* the pressure, *I* the identity tensor, *μ* the coefficient of viscosity, $\dot{\gamma }$ the shear rate, $\frac{d}{dt}$ the material time differentiation and (*u*, *v*) are the components of velocity. The equations governing the flow are given by

$$\frac{\partial u}{\partial x}+\frac{\partial v}{\partial y}=0,$$ (2)

$$\begin{array}{ll} \frac{\partial u}{\partial t}+u\frac{\partial u}{\partial x}+v\frac{\partial u}{\partial y}\;=\; & -\frac{1}{\rho_{f}}\frac{\partial p}{\partial x}+\frac{1}{\rho_{f}}\frac{\partial S_{xx}}{\partial x}+\frac{1}{\rho_{f}}\frac{\partial S_{xy}}{\partial y}-\frac{\sigma B_{0}^{2}u}{\rho_{f}}+\left(1-C_{0}\right)g\alpha \left(T-T_{0}\right)\\ & +\left(\frac{\rho_{p}-\rho_{f}}{\rho_{f}}\right)g\beta \left(C-C_{0}\right), \end{array}$$ (3)

$$\begin{array}{lll} \frac{\partial v}{\partial t}+u\frac{\partial v}{\partial x}+v\frac{\partial v}{\partial y} & = & -\frac{1}{\rho_{f}}\frac{\partial p}{\partial y}+\frac{1}{\rho_{f}}\frac{\partial S_{xy}}{\partial x}+\frac{1}{\rho_{f}}\frac{\partial S_{yy}}{\partial y}, \end{array}$$ (4)

$$\begin{array}{ccc} \frac{\partial T}{\partial t}+u\frac{\partial T}{\partial x}+v\frac{\partial T}{\partial y} & = & \alpha \left(\frac{\partial^{2}T}{\partial x^{2}}+\frac{\partial^{2}T}{\partial y^{2}}\right)+\frac{1}{\rho_{f}c_{f}}\left[S_{xx}\frac{\partial u}{\partial X}+S_{xy}\left(\frac{\partial u}{\partial y}+\frac{\partial v}{\partial x}\right)+S_{yy}\frac{\partial v}{\partial y}\right]\\ & & +\frac{1}{\rho_{f}c_{f}}\left(\frac{\partial q_{r}}{\partial y}+\frac{\partial q_{r}}{\partial x}\right)+\frac{\sigma B_{0}^{2}u^{2}}{\rho_{f}c_{f}}\\ & & +\tau \left[D_{B}\left(\frac{\partial C}{\partial x}\frac{dT}{dx}+\frac{\partial C}{\partial y}\frac{dT}{dy}\right)+\frac{D_{T}}{T_{m}}\left\{{\left(\frac{\partial T}{\partial x}\right)}^{2}+{\left(\frac{\partial T}{\partial y}\right)}^{2}\right\}\right], \end{array}$$ (5)

$$\frac{\partial C}{\partial t}+u\frac{\partial C}{\partial x}+v\frac{\partial C}{\partial y}=D_{B}\left(\frac{\partial^{2}C}{\partial x^{2}}+\frac{\partial^{2}C}{\partial y^{2}}\right)+\frac{D_{T}}{T_{m}}\left(\frac{\partial^{2}T}{\partial x^{2}}+\frac{\partial^{2}T}{\partial y^{2}}\right),$$ (6)

Radiative heat flux *q*_*r*_ is given by

$$q_{r}=-\frac{4\sigma^{*}}{3k^{*}}\frac{\partial T^{4}}{\partial y},$$ (7)

where σ* is the Stefan–Boltzmann constant having numerical value 1.380648 × 10^−23^*JK*^−1^ and k* is the mean absorption coefficient. We assume that the temperature difference within the flow is sufficiently small. Hence expanding *T*^4^about *T*_0_ and neglecting higher order terms one obtains

$$T^{4}≅4T_{0}^{3}T-3T_{0}^{4}.$$

The above expression and Eq (7) now yield

$$q_{r}=\frac{-16\sigma^{*}T_{0}^{3}}{3k^{*}}\frac{\partial T}{\partial y}.$$ (8)

The relevant boundary conditions are

$$u\pm \beta_{1}\frac{\mu }{1+\lambda_{1}}\left(1+\lambda_{2}\left(u\frac{\partial }{\partial x}+v\frac{\partial }{\partial y}\right)\right)\left(\frac{\partial u}{\partial y}+\frac{\partial v}{\partial x}\right)=0,\;\;\;at\;\;y=\pm \eta ,$$ (9)

T±β2∂T∂y=T1T0,C±β3∂C∂y=C1C0,aty=±η, (10)

$$\begin{array}{ll} {}[-\tau_{1}\frac{\partial^{3}}{\partial x^{3}} & +m_{1}\frac{\partial^{3}}{\partial x\partial t^{2}}+d\frac{\partial^{2}}{\partial x\partial t}]\eta =\frac{\partial S_{xx}}{\partial x}+\frac{\partial S_{xy}}{\partial y}-\rho_{f}\left(\frac{\partial u}{\partial t}+u\frac{\partial u}{\partial x}+v\frac{\partial u}{\partial y}\right)-\sigma B_{0}^{2}u\\ & -\left(1-C_{0}\right)\rho_{f}g\alpha \left(T-T_{0}\right)+(\rho_{p}-\rho_{f})g\beta \left(C-C_{0}\right),\;\text{at}\;\;\text{y}=\pm \eta . \end{array}$$ (11)

In the above equations *ρ*_*f*_ is the density of the nanofluid, *ν* the kinematic viscosity, *α* the thermal diffusivity, *σ* the thermal conductivity, *S*_*xx*_, *S*_*xy*_, *S*_*yy*_ the components of extra stress tensor, *D*_*B*_ the Brownian motion coefficient, *D*_*T*_ the thermophoretic diffusion coefficient and $\tau^{*}=\frac{{\left(\rho c\right)}_{p}}{{\left(\rho c\right)}_{f}}$ the ratio of effective heat capacity of nanoparticle material to heat capacity of fluid, *τ*_1_ the elastic tension, *m*_1_ the mass per unit area, *d*_1_ the coefficient of viscous damping, *β*_1_, *β*_2_, *β*_3_ the velocity, thermal and concentration slip parameters respectively, *T*_*m*_ the mean temperature, *T*_1_ and *C*_1_ the temperature and the concentration at the right wall respectively while *T*_0_ and *C*_0_ the temperature and the concentration at the left wall respectively.

Introducing the following non-dimensional quantities:

$$\begin{array}{ccc} u^{*} & = & \frac{u}{c},\;v^{*}=\frac{v}{c},\;x^{*}=\frac{x}{\lambda },\;y^{*}=\frac{y}{d_{1}},\;\beta_{i}^{*}=\frac{\beta_{i}}{d_{1}}\left(i=1,2,3\right),\;t^{*}=\frac{ct}{\lambda },\;\eta^{*}=\frac{\eta }{d_{1}},\;p^{*}=\frac{d_{1}^{2}p}{c\lambda \mu },\;K=\frac{k}{d^{2}},\\ \theta & = & \frac{T-T_{0}}{T_{1}-T_{0}},\;\phi =\frac{C-C_{0}}{C_{1}-C_{0}},\;Gr=\frac{\left(1-C_{0}\right)\rho_{f}g\alpha d^{2}\left(T_{1}-T_{0}\right)}{c\mu },\;Qr=\frac{\left(\rho_{c}-\rho_{f}\right)g\beta d^{2}\left(C_{1}-C_{0}\right)}{c\mu },\;\\ E_{1} & = & -\frac{\tau d_{1}^{3}}{\lambda^{3}\mu c},\;E_{2}=\frac{m_{1}d_{1}^{3}c}{\lambda^{3}\mu },\;E_{3}=\frac{dd_{1}^{3}}{\lambda^{2}\mu },\;Rn=\frac{16\sigma T_{0}^{3}}{3k\mu c_{f}},\;s_{ij}=\frac{d_{1}}{\mu c}S_{ij}^{*},\;\lambda_{2}^{*}=\frac{\lambda_{2}c}{d_{1}}.\; \end{array}$$ (12)

Eqs (3)–(6) after omitting asterisks and writing stream function *ψ*(*x*, *y*, *t*) by the definition [7]:

$$u=\frac{\partial \psi }{\partial y},\;\;\;\;v=-\delta \frac{\partial \psi }{\partial x},$$

become

$$\begin{array}{ccc} Re\left[\delta \frac{\partial^{2}\psi }{\partial t\partial y}+\delta \frac{\partial \psi }{\partial y}\frac{\partial^{2}\psi }{\partial x\partial y}-\delta \frac{\partial \psi }{\partial x}\frac{\partial^{2}\psi }{\partial y^{2}}\right] & = & -\frac{\partial p}{\partial x}+\delta \frac{\partial s_{xx}}{\partial x}+\frac{\partial s_{xy}}{\partial y}\\ & & +Gr\theta +Qr\phi -M^{2}u, \end{array}$$ (13)

$$Re\delta \left[-\delta^{2}\frac{\partial^{2}\psi }{\partial x\partial t}-\delta^{2}\frac{\partial \psi }{\partial y}\frac{\partial^{2}\psi }{\partial x^{2}}-\delta^{2}\frac{\partial^{2}\psi }{\partial x\partial y}\right]=-\frac{\partial p}{\partial y}+\delta^{2}\frac{\partial s_{xy}}{\partial x}+\delta \frac{\partial s_{yy}}{\partial y},$$ (14)

$$\begin{array}{ccc} Re\left[\delta \frac{\partial \theta }{\partial t}+u\delta \frac{\partial \theta }{\partial x}+v\frac{\partial \theta }{\partial y}\right] & = & Ec\left(\frac{1}{1+\lambda_{1}}\right)\left[\left(1+\lambda_{2}^{*}\delta \left(\frac{\partial \psi }{\partial y}\frac{\partial }{\partial x}-\frac{\partial \psi }{\partial x}\frac{\partial }{\partial y}\right)\right)\right.\\ & & \left.\left(4\delta^{2}{\left(\frac{\partial^{2}\psi }{\partial x\partial y}\right)}^{2}+{\left(-\delta^{2}\frac{\partial^{2}\psi }{\partial x^{2}}+\frac{\partial^{2}\psi }{\partial y^{2}}\right)}^{2}\right)\right]\\ & & +Rn\left(\delta \frac{\partial^{2}\theta }{\partial x\partial y}+\frac{\partial^{2}\theta }{\partial y^{2}}\right)+\frac{1}{\mathrm{Pr}}\left(\delta^{2}\frac{\partial^{2}\theta }{\partial x^{2}}+\frac{\partial^{2}\theta }{\partial y^{2}}\right)+EcM^{2}{\left(\frac{\partial \psi }{\partial y}\right)}^{2}\\ & & +Nb\left[\delta^{2}\frac{\partial \phi }{\partial x}\frac{\partial \theta }{\partial x}+\frac{\partial \phi }{\partial y}\frac{\partial \theta }{\partial y}\right]+Nt\left[{\left(\delta \frac{\partial \theta }{\partial x}\right)}^{2}+{\left(\frac{\partial \theta }{\partial y}\right)}^{2}\right], \end{array}$$ (15)

$$ReSc\left[\delta \frac{\partial \phi }{\partial t}+\delta \frac{\partial \psi }{\partial y}\frac{\partial \phi }{\partial x}-\delta \frac{\partial \psi }{\partial x}\frac{\partial \phi }{\partial y}\right]=\left(\delta^{2}\frac{\partial^{2}\phi }{\partial x^{2}}+\frac{\partial^{2}\phi }{\partial y^{2}}\right)+\frac{Nt}{Nb}\left(\delta^{2}\frac{\partial^{2}\theta }{\partial x^{2}}+\frac{\partial^{2}\theta }{\partial y^{2}}\right),$$ (16)

with the boundary conditions

$$\frac{\partial \psi }{\partial y}\pm \beta_{1}\frac{1}{1+\lambda_{1}}\left[1+\lambda_{2}^{*}\delta \right.\left.\left(\frac{\partial \psi }{\partial y}\frac{\partial }{\partial x}-\delta^{2}\frac{\partial \psi }{\partial x}\frac{\partial }{\partial y}\right)\right]\left(\frac{\partial^{2}\psi }{\partial y^{2}}-\delta \frac{\partial^{2}\psi }{\partial x^{2}}\right)=0,\;\;\;at\;\;y=\pm \eta ,$$ (17)

θ±β2∂θ∂y=10,ϕ±β3∂ϕ∂y=10,aty=±η, (18)

$$\left[E_{1}\frac{\partial^{3}}{\partial x^{3}}+E_{2}\frac{\partial^{3}}{\partial x\partial t^{2}}+E_{3}\frac{\partial^{2}}{\partial x\partial t}\right]\eta =\frac{1}{1+\lambda_{1}}\left(1+\delta \frac{\partial \psi }{\partial y}\frac{\partial }{\partial x}\right)\frac{\partial^{3}\psi }{\partial y^{3}}+Gr\theta +Qr\phi -M^{2}\frac{\partial \psi }{\partial y},\;\;at\;\;y=\pm \eta ,$$ (19)

where

$$\begin{array}{ccc} & & s_{{}_{xx}=}\frac{2\delta }{1+\lambda_{1}}\left[1+\lambda_{2}^{*}\delta \left(\frac{\partial \psi }{\partial y}\frac{\partial }{\partial x}-\frac{\partial \psi }{\partial x}\frac{\partial }{\partial y}\right)\right]\frac{\partial^{2}\psi }{\partial x\partial y},\\ & & s_{{}_{xy}=}\frac{1}{1+\lambda_{1}}\left[1+\lambda_{2}^{*}\delta \left(\frac{\partial \psi }{\partial y}\frac{\partial }{\partial x}-\frac{\partial \psi }{\partial x}\frac{\partial }{\partial y}\right)\right]\left(\frac{\partial^{2}\psi }{\partial y^{2}}-\delta^{2}\frac{\partial^{2}\psi }{\partial x^{2}}\right),\\ & & s_{{}_{yy}=-}\frac{2\delta }{1+\lambda_{1}}\left[1+\lambda_{2}^{*}\delta \left(\frac{\partial \psi }{\partial y}\frac{\partial }{\partial x}-\frac{\partial \psi }{\partial x}\frac{\partial }{\partial y}\right)\right]\frac{\partial^{2}\psi }{\partial x\partial y}, \end{array}$$

After employing long wavelength and low Reynolds number approximations [18] one has the following problems

$$\left(\frac{1}{1+\lambda_{1}}\right)\frac{\partial^{4}\psi }{\partial y^{4}}+Gr\frac{\partial \theta }{\partial y}+Qr\frac{\partial \phi }{\partial y}-M^{2}\frac{\partial^{2}\psi }{\partial y^{2}}=0,$$ (20)

$$\begin{array}{l} (1+Rn\text{Pr})\frac{\partial^{2}\theta }{\partial y^{2}}+Nb\text{Pr}\frac{\partial \theta }{\partial y}\frac{\partial \phi }{\partial y}+Nt\text{Pr}{\left(\frac{\partial \theta }{\partial y}\right)}^{2}\\ +Br\;\left[\frac{1}{1+\lambda_{1}}{\left(\frac{\partial^{2}\psi }{\partial y^{2}}\right)}^{2}+M^{2}{\left(\frac{\partial \psi }{\partial y}\right)}^{2}\right]=0, \end{array}$$ (21)

$$\frac{\partial^{2}\phi }{\partial y^{2}}+\frac{Nt}{Nb}\left(\frac{\partial^{2}\theta }{\partial y^{2}}\right)=0,$$ (22)

∂ψ∂y±β111+λ1∂2ψ∂y2=0,θ±β2∂θ∂y=10,ϕ±β3∂ϕ∂y=10,aty=±η, (23)

$$\left[E_{1}\frac{\partial^{3}}{\partial x^{3}}+E_{2}\frac{\partial^{3}}{\partial x\partial t^{2}}+E_{3}\frac{\partial^{2}}{\partial x\partial t}\right]\eta =\left(\frac{1}{1+\lambda_{1}}\right)\frac{\partial^{3}\psi }{\partial y^{3}}+Gr\theta +Qr\phi -M^{2}\frac{\partial \psi }{\partial y},\;\;at\;\;y=\pm \eta ,$$ (24)

where $\epsilon =\frac{a}{d_{1}}$ represents the amplitude ratio, $\delta =\frac{d_{1}}{\lambda }$ the wave number, $Nb=\frac{\tau^{*}D_{B}\left(C_{1-}C_{0}\right)}{\nu }$ and $Nt=\frac{\tau^{*}D_{T}\left(T_{1-}T_{0}\right)}{T_{m}\nu }$ the Brownian motion and thermophoresis parameters respectively, $Re=\frac{c\rho d_{1}}{\mu }$ the Reynolds number, $Sc=\frac{\nu }{D_{B}}$ the Schmidt number, $M=\sqrt{\frac{\sigma }{\mu }}B_{0}d_{1}$ the Hartman number, $Ec=\frac{c^{2}}{c_{f}\left(T_{1}-T_{0}\right)}$ the Eckert number, $\text{Pr}=\frac{\nu \rho c_{p}}{k}$ the Prandtl number, *Qr* the local nanoparticle Grashoff number and *Br* = Pr*Ec* the Brinkman number, *Pr* the Prandtl number and *Ec* Eckert number. Heat transfer coefficient in non-dimensional form is given by

$$Z={\left.\eta_{x}\frac{\partial \theta }{\partial y}\right|}_{y=\eta }.$$ (25)

## 3 Discussion

The purpose of this portion is to predict the behaviors of velocity, temperature, nanoparticle concentration and heat transfer rate under the impact of emerging parameters. Hence the graphical results are obtained numerically through NDSolve in Mathematica. The relevant physical explanations are presented in this section.

### 3.1 Velocity profile

**Fig 2:**
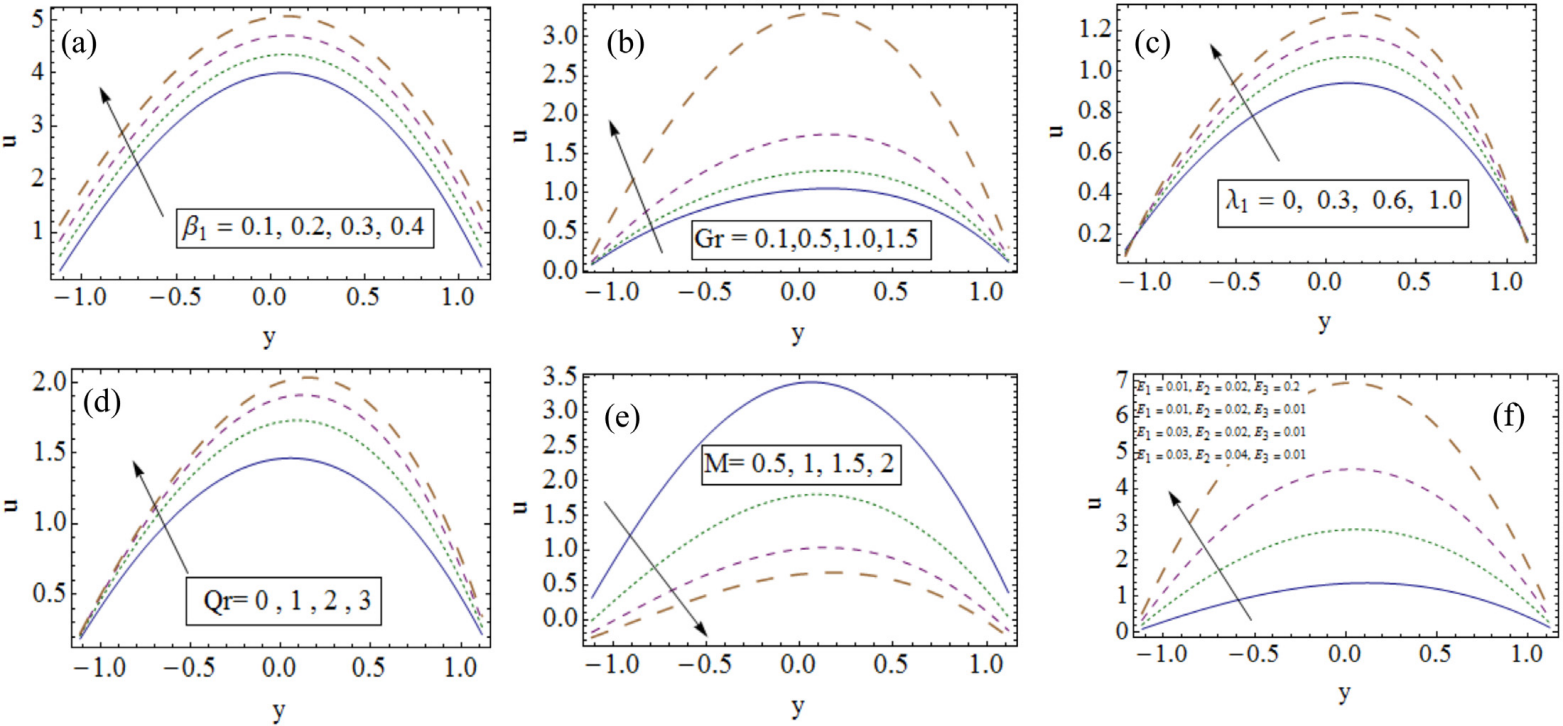
Influences of different parameters on velocity when (a). *x* = 0.2, *t* = 0.1, Pr = 1.5, *Nt* = 0.1, *Nb* = 0.1, *Br* = 1.7, *Qr* = 1, *Gr* = 1.5, *ϵ* = 0.2, *M* = 0.2, *E*_1_ = 0.01, *E*_2_ = 0.02, *E*_3_ = 0.01, *β*_2_ = 0.1, *β*_3_ = 0.1, *λ*_1_ = 1, *Rn* = 1. (b). *x* = 0.2, *t* = 0.1, Pr = 0.8, *Nt* = 0.3, *Nb* = 0.2, *Br* = 1.7, *Qr* = 1, *ϵ* = 0.2, *M* = 1, *E*_1_ = 0.01, *E*_2_ = 0.02, *E*_3_ = 0.01, *β*_1_ = 0.1, *β*_2_ = 0.1, *β*_3_ = 0.1, *λ*_1_ = 1, *Rn* = 1. (*c*). *x* = 0.2, *t* = 0.1, Pr = 0.8, *Nt* = 0.3, *Nb* = 0.2, *Nt* = 1.7, *Qr* = 1, *Gr* = 0.5, *ϵ* = 0.2, *M* = 1, *E*_1_ = 0.01, *E*_2_ = 0.02, *E*_3_ = 0.01, *β*_1_ = 0.1, *β*_2_ = 0.1, *β*_3_ = 0.1, *Rn* = 1. (*d*). *x* = 0.2, *t* = 0.1, *Pr* = 1, *Nt* = 0.1, *Nb* = 0.1, *Br* = 1.7, *Gr* = 1.5, *ϵ* = 0.2, *M* = 0.2, *E*_1_ = 0.01, *E*_2_ = 0.02, *E*_3_ = 0.01, *β*_1_ = 0.1, *β*_2_ = 0.1, *β*_3_ = 0.1, *λ*_1_ = 0.1, *Rn* = 1. (*e*). *x* = 0.2, *t* = 0.1, *Pr* = 1, *Nt* = 0.1, *Nb* = 0.1, *Br* = 0.5, *Qr* = 0.3, *Gr* = 1, *ϵ* = 0.2, *M* = 0.2, *β*_1_ = 0.1, *β*_2_ = 0.1, *β*_3_ = 0.1, *λ*_1_ = 1, *Rn* = 1. (*f*). *x* = 0.2, *t* = 0.1, Pr = 1.5, *Nt* = 0.1, *Nb* = 0.1, *Br* = 1.7, *Qr* = 1, *Gr* = 1.5, *ϵ* = 0.2, *M* = 0.2, *β*_1_ = 0.1, *β*_2_ = 0.1, *β*_3_ = 0.1, *λ*_1_ = 1, *Rn* = 1.

Fig 2 represents the impact of various parameters on velocity. In (Fig 2(a)) it is observed that for larger velocity slip parameter *β*_1_, the velocity increases. As fluid slip is the deviation in the angle at which the fluid leaves the channel. Therefore an increase in *β*_1_ causes non-uniform velocity distribution inside the channel (see Fig 2(a)). The effect is useful in determining the accurate estimation of energy transfer between the channel and the fluid. Similar result has been obtained by Hayat et. al [13] in their study for nanofluids. The reason behind the increasing behavior of *β*_1_ is that the resistance is reduced due to slip hence velocity increases. Increasing Grashoff number *Gr* decreases drag forces and hence velocity profile increases (see Fig 2(b)). It is seen that velocity is an increasing function of Jeffrey fluid parameter *λ*_1_ and local nanoparticle Grashoff number *Qr* (see Fig 2(c) and 2(d)). Magnetic field applied in transverse direction acts as a retarding force for the fluid flow and thus the velocity profile decreases for increasing values of Hartman number *M* (see Fig 2(e)). Due to elasticity of flexible walls the velocity profile shows increasing behavior for larger *E*_1_ and *E*_2_ while velocity profile decreases upon enhancement in *E*_3_ due to its viscous damping effect (see Fig 2(f)). For reliability the results obtained for wall parameters can be compared with the previous analysis of Hina et al. [11] for curved channel and Hayat et al. [13] for planer channel.

### 3.2 Temperature profile

**Fig 3:**
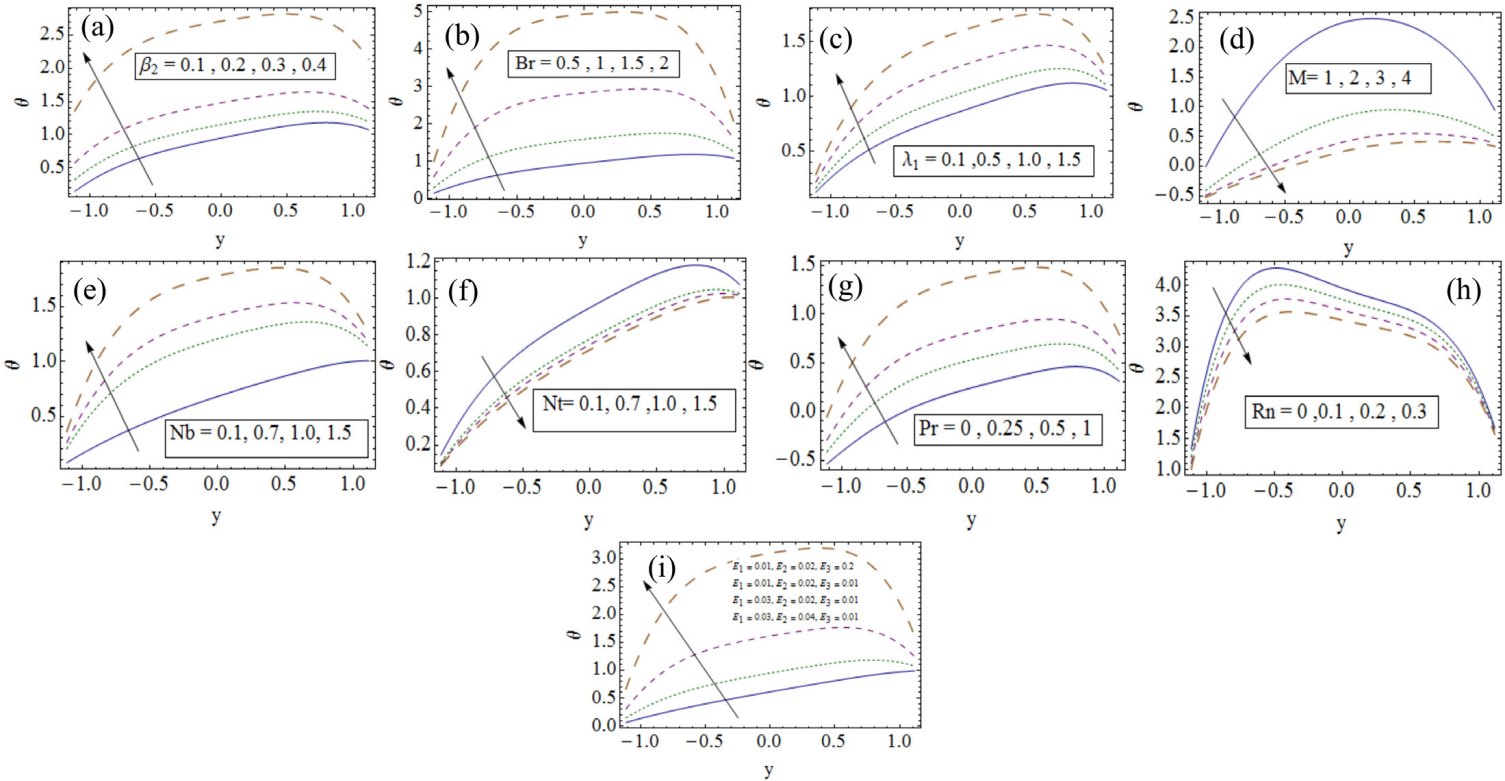
Influences of different parameters on temperature profile when (a). *x* = *ϵ* = 0.2, *t* = 0.1, *Rn* = 1, *Gr* = 1, *Qr* = 0.3, *λ*_1_ = 1, *Br* = 0.5, *Pr* = 1, *Nt* = *Nb* = 0.1, *M* = 0.2, *β*_1_ = *β*_3_ = 0.1, *E*_1_ = *E*_3_ = 0.01, *E*_2_ = 0.02. (b). *x* = *ϵ* = 0.2, *t* = 0.1, *λ*_1_ = 0.3, *Rn* = 1, *Gr* = 1.5, *Qr* = 1, *Pr* = 1, *Nt* = *Nb* = 0.1, *M* = 0.2, *β*_1_ = *β*_2_ = *β*_3_ = 0.1, *E*_1_ = *E*_3_ = 0.01, *E*_2_ = 0.02. (c). *x* = *ϵ* = 0.2, *t* = 0.1, *Rn* = 1, *Gr* = 1.5, *Qr* = 1, *Br* = 0.5, *Pr* = 1, *Nt* = *Nb* = 0.1, *M* = 0.2, *β*_1_ = *β*_2_ = *β*_3_ = 0.1, *E*_1_ = *E*_3_ = 0.01, *E*_2_ = 0.02. (d). *x* = *ϵ* = 0.2, *t* = 0.1, *Gr* = 1, *Qr* = 1, *Rn* = 1, *Gr* = 1.5, *Qr* = 1, *λ*_1_ = 0.3, *Br* = 0.5, *Pr* = 1, *Nt* = *Nb* = 0.1, *M* = 0.2, *β*_1_ = *β*_3_ = 0.1, *E*_1_ = *E*_3_ = 0.01, *E*_2_ = 0.02. (e). *x* = *ϵ* = 0.2, *t* = 0.1, *Rn* = 1, *Gr* = 1.5, *Qr* = 0.3, *λ*_1_ = 1, *Br* = 0.5, *Pr* = 1, *Nt* = 0.1, *M* = 0.5, *β*_1_ = *β*_2_ = *β*_3_ = 0.1, *E*_1_ = *E*_3_ = 0.01, *E*_2_ = 0.02. (f). *x* = *ϵ* = 0.2, *t* = 0.1, *Rn* = 1, *Gr* = 1, *Qr* = 0.3, *λ*_1_ = 1, *Br* = 0.5, *Pr* = 1, *Nb* = 0.1, *M* = 0.2, *β*_1_ = *β*_2_ = *β*_3_ = 0.1, *E*_1_ = *E*_3_ = 0.01, *E*_2_ = 0.02. (g). *x* = *ϵ* = 0.2, *t* = 0.1, *Rn* = 2, *Gr* = 1.5, *Qr* = 0.3, *λ*_1_ = 1, *Br* = 1.7, *Nt* = 0.1, *Nb* = 1, *M* = 0.2, *β*_1_ = *β*_2_ = *β*_3_ = 0.1, *E*_1_ = *E*_3_ = 0.01, *E*_2_ = 0.02. (h). *x* = *ϵ* = 0.2, *t* = 0.1, *Gr* = 1.5, *Qr* = 0.3, *λ*_1_ = 1, *Br* = 1.7, *Pr* = 1, *Nt* = 3, *Nb* = 2, *M* = 0.2, *β*_1_ = *β*_2_ = *β*_3_ = 0.1, *E*_1_ = *E*_3_ = 0.01, *E*_2_ = 0.02. (i). *x* = *ϵ* = 0.2, *t* = 0.1, *Gr* = 1, *Qr* = 1, *Rn* = 1, *Gr* = 1, *Qr* = 0.3, *λ*_1_ = 1, *Br* = 0.5, *Pr* = 1, *Nt* = *Nb* = 0.1, *M* = 0.2, *β*_1_ = *β*_2_ = *β*_3_ = 0.1.

Fig 3 displays the impacts of various parameters on temperature profile. It is observed from Fig 3(a) that temperature profile increases for larger thermal slip parameter *β*_2_. Fig 3(b) is plotted to see the variation of Brinkman number *Br* on temperature. Brinkman number arises due to viscous dissipation which enhances the temperature. It is reasonable to say that rise in temperature is produced by the stress-reversal process that develops with an increase in *β*_2_ (see Fig 3(b)). The similar observation for Carreau fluid have been reported by Vajravelu et al. [15]. By increasing Jeffrey nanofluid parameter the temperature profile enhances (see Fig 3(c)). Magnetic force acts as a retarding force and so it slows down the motion of fluid particles. As a result the kinetic energy decreases and thus temperature decreases by increasing Hartman number *M* (see Fig 3(d)). Here the obtained numerical results are found well matched with the perturbed results by Hayat et al. [7]. Temperature is more for increasing values of Brownian motion parameter *Nb* because it makes the motion of nanoparticles stronger (see Fig 3(e)). However it decreases for increasing values of thermophoresis parameter *Nt* (see Fig 3(f)). Since nanoparticles possess strong thermal gradients producing nonlinear dependence of the drift velocity on the applied gradient for large *Nt*. Thus nonlinear thermophoresis can cause contradictory results between thermophoretic and numerical analysis. That is why the numerical results develops the nonlinear temperature distribution with *Nt* similar to the study of Hayat et al. [13]. Temperature shows increasing behavior for larger Prandtl number Pr (see Fig 3(g)). It is in view of an increase in specific heat. An increase in thermal radiation parameter *Rn* decreases the temperature (as noticed from Fig 3(h)). Temperature is the increasing functions of *E*_1_ and *E*_2_ due to elastance of wall while it decreases for *E*_3_ as *E*_3_ shows oscillatory resistance (see Fig 3(i)).

### 3.3 Nanoparticle concentration profile

**Fig 4:**
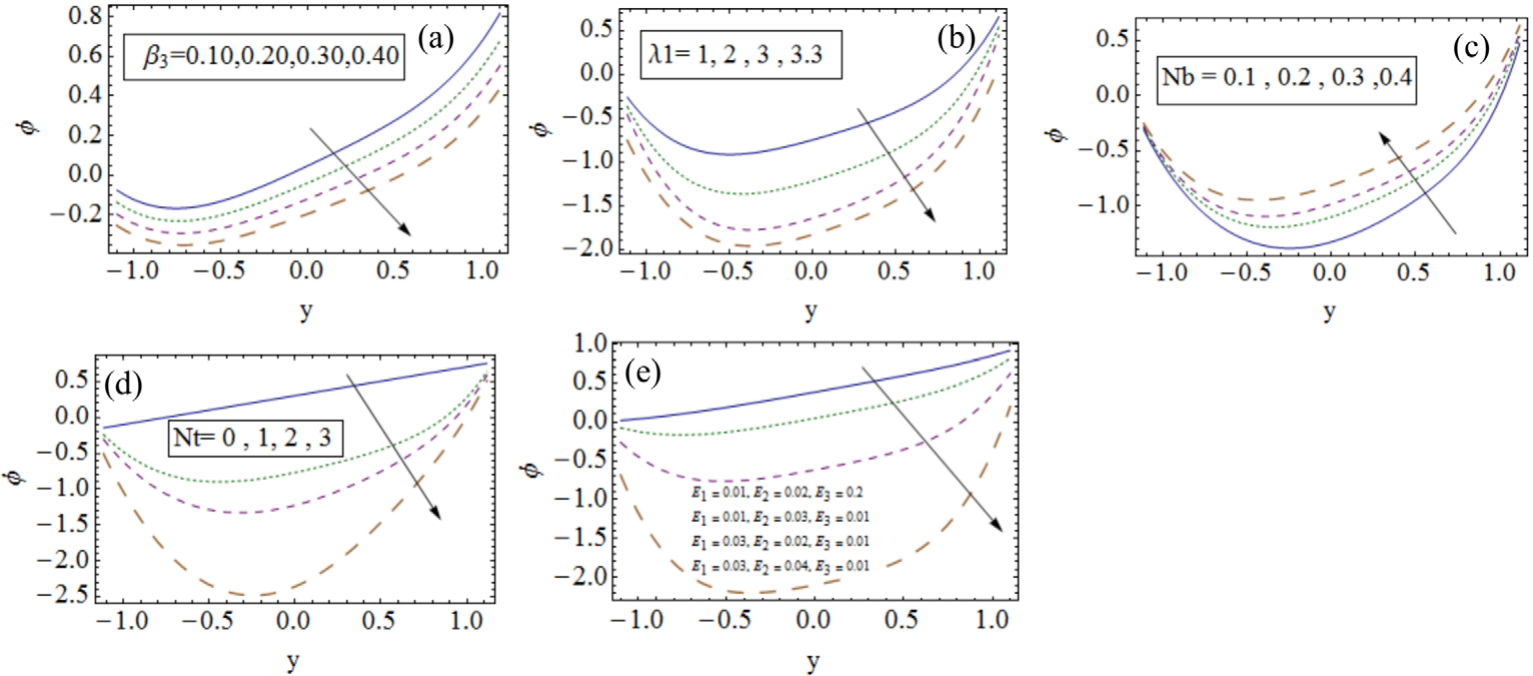
Influences of different parameters on nanoparticle concentration profile when (a). *x* = *ϵ* = 0.2, *t* = 0.1, *Gr* = 1, *Qr* = 0.3, *Br* = 0.5, *Nt* = *Nb* = 0.1, *Pr* = 1, *M* = 0.2, *λ*_1_ = 1, *Rn* = 1, *β*_1_ = *β*_2_ = 0.1, *E*_1_ = *E*_3_ = 0.01, *E*_2_ = 0.02. (*b*). *x* = *ϵ* = 0.2, *t* = 0.1, *Gr* = 0.8, *Qr* = 1, *Pr* = 1, *Br* = 1.7, *M* = 0.2, *Rn* = 1, *β*_1_ = *β*_2_ = *β*_3_ = 0.1, *E*_1_ = *E*_3_ = 0.01, *E*_2_ = 0.02. (*c*). *x* = *ϵ* = 0.2, *t* = 0.1, *Gr* = 0.8, *Qr* = 0.3, *Br* = 1.7, *Nt* = 0.1, *Pr* = 1, *M* = 0.2, *λ*_1_ = 1, *Rn* = 1, *β*_1_ = *β*_2_ = *β*_3_ = 0.1, *E*_1_ = *E*_3_ = 0.01, *E*_2_ = 0.02. (*d*). *x* = *ϵ* = 0.2, *t* = 0.1, *Gr* = 1, *Br* = 1.7, *Qr* = 0.3, *λ*_1_ = 1, *Rn* = 1, *Nb* = 0.3, *Pr* = 1, *M* = 0.2, *β*_1_ = *β*_2_ = *β*_3_ = 0.1, *E*_1_ = *E*_3_ = 0.01, *E*_2_ = 0.02. (*e*). *x* = *ϵ* = 0.2, *t* = 0.1, *β*_1_ = *β*_2_ = *β*_3_ = 0.1, *Gr* = 1, *Qr* = 0.3, *Br* = 0.5, *Nt* = *Nb* = 0.1, *Pr* = 1, *M* = 0.2, *λ*_1_ = 1, *Rn* = 1, *β*_1_ = *β*_2_ = *β*_3_ = 0.1.

Fig 4 illustrates the influences of various parameters on nanoparticles concentration profile. It is noticed that by increasing values of concentration slip parameter *β*_3_ the nanoparticle concentration decreases (see Fig 4(a)). Fig 4(b) shows that concentration is a decreasing function of Jeffrey nanofluid parameter *λ*_1_. An increasing behavior of nanoparticle concentration is seen with Brownian motion parameter *Nb* (see Fig 4(c)). Also nanoparticle concentration profile is decreasing function of thermophoresis parameter *Nt* (see Fig 4(d)). Fig 4(e) depicts that when *E*_1_ and *E*_2_ increased then concentration profile decreases while it increases for *E*_3_.

### 3.4 Heat transfer coefficient

**Fig 5:**
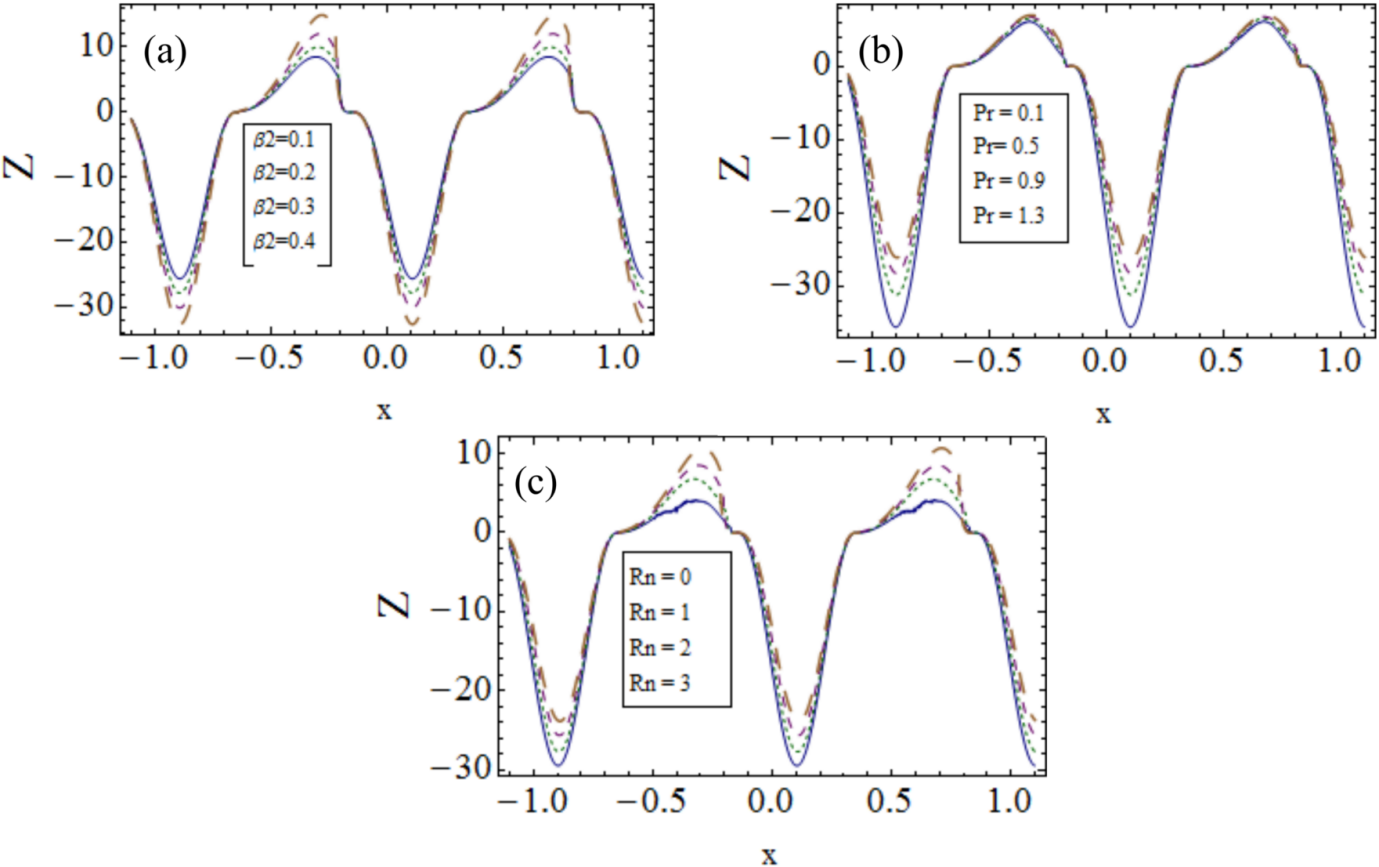
Influences of different parameters on heat transfer coefficient Z(x) when (a). *ϵ* = 0.2, *t* = 0.1, *Rn* = 2, *Gr* = 0.3, *Qr* = 1, *λ*_1_ = 1, *Br* = 0.5, *Pr* = 1, *Nt* = *Nb* = 1, *M* = 2, *β*_1_ = *β*_3_ = 0.1, *E*_1_ = 0.1, *E*_3_ = 0.01, *E*_2_ = 0.2. (b). *ϵ* = 0.2, *t* = 0.1, *λ*_1_ = 1, *Rn* = 2, *Gr* = 0.3, *Qr* = 1, *Nt* = *Nb* = 1, *M* = 2, *β*_1_ = *β*_2_ = *β*_3_ = 0.1, *E*_1_ = 0.1, *E*_3_ = 0.01, *E*_2_ = 0.2. (c). *ϵ* = 0.2, *t* = 0.1, *Gr* = 0.3, *Qr* = 1, *Br* = 0.4, *Nt* = *Nb* = 0.1, *M* = 2, *β*_1_ = *β*_2_ = *β*_3_ = 0.1, *E*_1_ = 0.1, *E*_3_ = 0.01, *E*_2_ = 0.2.

Fig 5 describes the influence of various emerging parameters on *Z*. It has been observed that because of successive contraction and relaxation of peristaltic walls the heat transfer coefficient shows oscillatory behavior. For thermal slip parameter *β*_2_ the absolute value of *Z* decreases (see Fig 5(a)). Heat transfer is an increasing function of Prandtl number Pr (see Fig 5(b)). Also larger radiation parameter *Rn* enhances the heat transfer coefficient *Z* (see Fig 5(c)).

## 4 Concluding remarks

Analysis is performed for the peristaltic flow of Jeffrey nanofluid in a channel with compliant walls partial slip and mixed convection. The major observations are listed below:

- Behavior of *λ*_1_ on velocity and temperature is similar.
- Concentration and temperature have reverse effect for *λ*_1_.
- Grashoff number *Gr* and local nanoparticle Grashoff number *Qr* enhance the velocity.
- Qualitatively the radiation *Rn* and thermophoresis *Nt* parameters have same effects on temperature.
- Brownian motion parameter enhances the temperature.
- Effects of *E*_1_ and *E*_2_ on velocity are similar. However the impact of *E*_3_ on velocity is opposite to that of *E*_1_ and *E*_2_.
- Behaviors of *β*_1_ on velocity and *β*_2_ on temperature are similar. However concentration decreases upon increasing slip parameter *β*_3_.
- Hartman number *M* reduces the temperature and velocity.
- Both temperature and heat transfer coefficient are increased for Prandtl number Pr.
- Concentration profile is decreasing function of *E*_1_ and *E*_2_. However the role of *E*_3_ on concentration is reverse to that of *E*_1_ and *E*_2_.
